# Government environmental attention and urban ecosystems metabolic efficiency: Does the digital economy matter

**DOI:** 10.1371/journal.pone.0332993

**Published:** 2025-09-24

**Authors:** Xiangmin He, Shenrun Yan, Jingyun Miao, Weilin Zeng

**Affiliations:** 1 School of Economics and Trade, Nanchang Institute of Technology, Nanchang, China; 2 School of Business Administration, Nanchang Institute of Technology, Nanchang, China; Tsinghua University, CHINA

## Abstract

Metabolic efficiency serves as a critical indicator of the operational quality of urban ecosystems. This study utilizes panel data from Chinese cities and applies the spatial Durbin model to examine the impact of government environmental attention (GEA) on the metabolic efficiency of urban ecosystems (UME). The findings indicate that GEA significantly promotes UME and exhibits spatial spillover effects. Moreover, GEA demonstrates notable heterogeneity in its influence on UME. Specifically, it exerts a more pronounced improvement effect in mid-sized and western cities, resource-based cities, and cities with relatively lower levels of economic development. Using the dynamic panel threshold regression model, this study further reveals that GEA has a significant nonlinear impact on UME, with digital economy development serving as the threshold variable. Once the level of digital economy development surpasses the threshold, the role of GEA becomes even more prominent. This research provides valuable decision-making references for developing countries aiming to promote the sustainable development of cities.

## 1. Introduction

Cities, as human settlements, require substantial resources for their operation and generate significant waste.Consequently, they can be conceptualized as ecosystems with metabolic functions. Wolman (1965) [1] pioneered the concept of urban metabolism in the context of urban ecological studies in the United States. He defined urban metabolism as the process by which cities acquire materials and energy inputs and expel waste. He posited that cities function as organic entities with metabolic capabilities. By drawing parallels between natural and urban ecosystems, he proposed that urban ecosystems sustain themselves by exchanging matter and energy with natural systems. Since then, research on urban metabolism has proliferated. The concept has become more comprehensive and multifaceted, reflecting the expansion of research areas and changes in urban forms. Decker et al.(2000) [2] described cities as organisms that consume building materials, fuels, and water, converting them into built environments, human biomass, and waste. Kennedy et al.(2011) [3] viewed urban metabolism as the flow of matter and energy within cities, influencing social development and the natural environment. Baccini (1997) [4] argued that the metabolism of modern cities—encompassing resource utilization, transformation, and disposal—is shaped by societal characteristics and global economic participation, primarily driven by fossil fuels such as oil and natural gas.

Urban metabolism, as a critical metric for assessing cities’ contributions to sustainable development [5], has garnered increasing scholarly attention. Recent studies have explored strategies to enhance UME. D’Amico et al.(2021) [6] found that digital technologies offer policymakers, urban managers, and planners valuable tools for collecting, monitoring, analyzing, and evaluating the circularity of environmental, social, and economic resources, thereby improving UME. Liao (2024) [7] argued that new urbanization initiatives have enhanced UME by promoting urban-rural integration and regional coordination, curbing unplanned urban expansion and energy misallocation, facilitating urban-rural population mobility, and fostering green innovation collaboration. Han et al.(2024) [8] demonstrated that green finance significantly boosts UME, contributing positively to urban sustainability. Liao et al. (2024) [9] further proposed that smart city construction can enhance UME through technological innovation, industrial upgrading, energy conservation, and optimized spatial structure. Generally speaking, these findings underscore the irreplaceable role of improving UME in advancing sustainable urban development.

Government attention is a behavioral process wherein decision-making entities selectively prioritize significant information while disregarding other data. The greater the GEA, the more emphasis local governments place on environmental protection, thereby enhancing their guiding role in environmental governance [10]. Key terms such as environmental protection, green development, and dual-carbon targets frequently appear in recent Chinese government Work Reports and Five-Year Plans, signaling that environmental performance has become a paramount concern for the Chinese government relative to economic growth [11]. Dong and Wang (2021) [12] discovered that heightened environmental awareness among both the public and the government significantly boosts green technology innovation and facilitates its transformation towards greener technologies. Farooq et al. (2021) [13] posits that governmental environmental attention critically influences corporate real investment. Consequently, GEA can impact resource utilization, conversion, and waste management in urban ecosystems, thereby affecting metabolic efficiency. However, to our knowledge, no prior literature has addressed this issue; this paper aims to fill this gap by examining the mechanisms and effects of GEA on the UME using spatial dynamic Durbin models and dynamic panel threshold regression models.

Compared with the existing literature, the contributions of this paper are as follows: (1) This study systematically investigates the influence mechanism and effect of GEA on UME. It fills a gap in the existing literature by addressing the underexplored impact of informal environmental regulations on UME, thereby enriching the research on the factors influencing UME and offering a reference for government decision-making departments to enhance UME through informal environmental regulations. (2) By employing text analysis methods, we quantify the environmental attention of local governments and connect it to UME. This elucidates the impact process of government policies on urban operations and provides a novel micro-level explanation for the role of local governments in developing countries regarding economic growth and social governance.(3)All existing literature on urban metabolic efficiency has neglected potential spatial spillover effects between cities, which may introduce biases into the estimation results. To address this gap, this study employs the Spatial Durbin Model (SDM) to account for such spatial interdependencies, thereby improving the accuracy of the estimation and offering empirical support for enhancing inter-city collaboration. (4)By considering the development of the digital economy as the threshold variable, we employ the dynamic panel threshold model to investigate the nonlinear impact of GEA on UME. The dynamic panel threshold model is capable of more precisely capturing the intricate relationships among variables, thereby facilitating a deeper understanding of the relationship between GEA and UME, including the specific level of digital economic development at which an increase in GEA leads to either an increase or decrease in UME. This model is robust to issues such as heteroscedasticity and autocorrelation. Utilizing the dynamic panel threshold model not only provides a powerful analytical tool for examining the nonlinear effect of GEA on UME but also offers valuable insights for policymakers in formulating and executing policies aimed at enhancing UME and promoting urban sustainable development.The remainder of this paper is structured as follows: Section 2 presents the theoretical hypotheses, outlining the three core hypotheses of this study. Section 3 details the research design, including the model specification, variable definitions, and data sources. Section 4 reports the empirical results, covering the regression analysis of the basic model, robustness tests, and heterogeneity analysis. Finally, Section 5 provides a discussion, conclusion and policy implications.

## 2. Theoretical hypothesis

According to the attention theory, attention involves the allocation of time, energy, and resources by a decision-making entity to a specific domain, issue, or space for conducting concentrated resource allocation activities related to that domain [14]. In this context, public decision-makers focus on a particular field, which is fundamentally a resource allocation activity. Once a decision-making entity prioritizes an issue within a field, it implies the need for corresponding resource allocations to achieve the decision-making objectives associated with that field [15,16]. The direction of governmental attention determines its decision-making behavior [17]. When government departments direct their focus toward a specific issue, it signifies that the matter has garnered governmental attention and suggests that appropriate actions are likely to follow.. Municipal governments’ environmental concern indicates their prioritization of environmental protection issues, thereby influencing relevant decisions, which can be observed in government work reports and other official documents.

Firstly, when the government allocates greater resources and policy attention to environmental protection, cities are likely to enhance their environmental governance. The government will implement more stringent environmental regulations, strengthen legislative frameworks, and refine environmental standards. Furthermore, to communicate its policy intentions more effectively, the government will consistently emphasize green, low-carbon, and environmentally sustainable policies through various channels, aiming to reduce information asymmetry between governmental bodies and market participants. For example, the government may demonstrate its commitment to environmental protection through formal communication channels, such as official reports and press conferences. Upon receiving these intensified policy signals, market entities may be inclined to adopt more proactive environmental measures to avoid regulatory penalties or enhance their corporate reputation. Concurrently, the government may encourage the adoption of environmentally friendly technologies—such as renewable energy systems and resource-saving technologies—which can improve urban resource utilization efficiency, minimize resource waste [18], and reduce undesirable outputs such as wastewater and emissions, thereby contributing to enhanced UME [19].

Secondly, when the government allocates more resources and policy attention to environmental protection, it will disseminate environmental information and knowledge to the public through diverse channels, such as advertisements, educational initiatives, and public relations campaigns. Consequently, public environmental awareness is expected to increase [20], leading to greater participation in environmental protection activities [18]. Enhanced environmental awareness and active public involvement can contribute to reduced resource consumption in urban areas [21], as well as a decrease in undesirable outputs [22].Thirdly, when the government allocates greater resources and policy attention to environmental protection, it signifies that environmental sustainability has become a strategic priority. Furthermore, the implementation and coordination of diverse regional environmental policies emerging from this heightened focus require effective governmental leadership, which facilitates the concentration of regional resources within the environmental sector. This entails that the government will direct various forms of capital—such as green credit facilities and dedicated funding programs—toward supporting environmentally responsible market entities through mechanisms including interest rate adjustments or other financial instruments [23]. Such interventions enable these entities to undertake green transformations, thereby reducing resource consumption, curbing pollution emissions, increasing corporate green output, and ultimately contributing to the enhancement of UME.

Finally, when the government allocates greater resources and policy attention to environmental protection, it sends a positive signal from the perspectives of market expectations and market signals, indicating that the local government places significant emphasis on environmental sustainability. This clarity facilitates the definition of development directions and objectives for green technological innovation. Increased governmental commitment to environmental protection enhances market entities’ awareness and understanding of ecological responsibilities, thereby fostering a favorable investment environment and strengthening confidence in the outcomes of green technological innovation. More importantly, the strategic prioritization of environmental protection encourages the reallocation of critical innovative resources—such as capital and talent—toward green technological innovation. It also guides market participants to invest more resources across all stages of the innovation chain, including green product design, green technology R&D, and environmentally sustainable production and manufacturing, thus promoting overall green technological innovation. Green technological innovation enables various economic actors within a city to conserve resources more effectively, increase resource marginal output, reduce resource utilization costs, and enhance overall resource efficiency [24]. Additionally, it contributes to the reduction of undesirable outputs [25], further improving UME.Therefore, this paper proposes the following research hypothesis:

H1: GEA positively contributes to enhancing UME.

The impact of the digital economy on the relationship between GEA and UME can be demonstrated in several aspects. Firstly, the advancement of the digital economy strengthens the positive role of GEA in environmental regulation, thereby further enhancing the improvement of UME. With the support of digital technology, governments can more effectively communicate their commitment to environmental protection to market entities and the public, thereby enabling businesses to make timely and targeted adjustments in alignment with policy objectives. The development of the digital economy is rooted in the application of digital technologies, which possess inherent green characteristics. These technologies can drive the advancement of clean production techniques and equipment, reduce environmental costs during production, lower pollutant emissions and energy consumption, improve economic efficiency, and mitigate environmental degradation. Through the aid of digital tools, critical resources such as capital, labor, and energy can be more efficiently directed toward low-carbon and environmentally sustainable industries, thus accelerating industrial structural upgrading. For enterprises themselves, the digital economy offers a source of technological innovation that enriches business models and operational content, ultimately fostering more sustainable enterprise development [26,27].

Secondly, From the perspective of public participation in environmental protection, the convenience, penetration, and extensive coverage enabled by digital economy development allow the public to more easily access environmental education, obtain environmental information, and engage in environmental supervision at a lower cost and with greater efficiency [28]. From the government management perspective, adopting digital technologies and e-government service models enhances the capacity and efficiency of governments for environmental governance, particularly at the grassroots level. Environmental governance costs for government departments but also broadens governance pathways and stimulates the willingness of environmental regulatory agencies to enforce regulations [29]. Through the collaborative efforts of government management and public supervision, enterprises are more motivated to conserve energy and reduce emissions, water consumption, and pollution. Additionally, public awareness of environmental protection is strengthened, and green consumption experiences substantial growth, all of which contribute to the enhancement of UME.

Thirdly, the development of the digital economy facilitates the acquisition of market information, mitigates the risk of information asymmetry, enhances the alignment between green credit, special funds, and relevant projects, and reduces the investment threshold for green financial products. Moreover, through data processing and analysis, the digital economy enables more precise identification and assessment of risks associated with green financial instruments, thereby allowing for refined risk management. This mechanism prevents certain enterprises from misappropriating green funds and special credits under the guise of green financing. For financial institutions, big data technology enhances the quality of green resources by organizing and categorizing large volumes of information, addressing the issue of information asymmetry between potential green investors and financial institutions. It also encourages the development of tiered financial products tailored to diverse investor needs, improves product design, and supports continuous innovation in green financial offerings. Consequently, this leads to increased efficiency in the utilization of green credit and special funds and further promotes UME.

Finally, the development of the digital economy enhances the promotion effect of GEA on green technology innovation, thereby improving UME. Specifically, the digital economy facilitates domestic and international information exchange, enabling R&D personnel to more easily access cutting-edge research progress, relevant data, and comprehensive research overviews. It enriches the research foundation and increases the likelihood of successful green innovation [30]. Indirectly, the digital economy provides greater opportunities for green industries to export to foreign markets, expanding market potential and driving green technology innovation [31]. Furthermore, digital finance, a critical dimension of the digital economy, alleviates financing constraints for SMEs, thereby fostering green technology innovation [32].

However, the influence of the digital economy on the relationship between GEA and UME is likely to be non-linear. Specifically, when the development level of the digital economy is relatively low, its limited capacity constrains the role of GEA in enhancing UME through mechanisms such as strengthening environmental regulation, promoting green technological innovation, facilitating industrial structure upgrading, and optimizing resource allocation. Conversely, once the digital economy reaches a higher level of development, it significantly amplifies the role of GEA in improving UME via the pathways above. Based on this reasoning, we propose Hypothesis 2.

**Fig 1:**
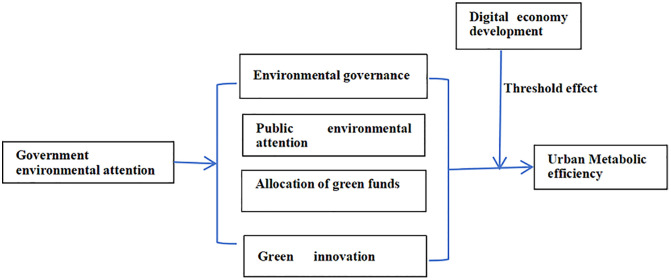
Theoretical framework diagram.

H2: The development of the digital economy exerts a threshold effect on the relationship between GEA and UME. When the development level of the digital economy surpasses a certain threshold, GEA exhibits a more pronounced positive impact on UME. Fig 1 illustrates the theoretical framework.

## 3. Research design

### 3.1. Model

#### 3.1.1. Spatial Durbin model.

Given that the utilization, transformation, and waste treatment of resources among cities may exhibit spatial interaction effects, we employ the dynamic spatial Durbin model for empirical testing. This model accounts for the spatial spillover effects of the dependent variable, thereby providing greater explanatory power than traditional panel data models. The specific formulation of the model is as follows:


$$UME_{it}=\rho WUME_{it}+\beta_{1}GEA_{it}+\theta_{1}WGEA_{it}+\sum \vartheta_{j}X_{kt}+W\sum \gamma_{k}X_{kt}+\mu_{i}+\delta_{t}+\varepsilon_{it}$$ (1)

In Equation (1), UMEit denotes the metabolic efficiency of urban ecosystem i in year t, while GEA reflects the government’s environmental concern. ρrepresents the spatial lag coefficient. X is a set of control variables. Drawing on existing literature, we include urban economic development level (GDP), urbanization level (Urb), financial development (FID), foreign direct investment (FDI), and fiscal intervention as control variables. W is the spatial weight matrix. We consider two types of weights: one is the standard spatial adjacency weight, and the other is the spatial economic connection weight. Given the actual conditions of urban economic development, we adopt the spatial economic connection weight matrix for analysis, with its calculation method presented in Equation (2).


$$W=\{\begin{array}{c} {}*20c1/\left|fgdp_{rp}\right|\left(r\neq p\right)\\ 0\left(r=p\right) \end{array}$$ (2)

$\left|fgdp_{rp}\right|$ represents the regional per capita GDP gap between cities r and p.

#### 3.1.2. Dynamic panel threshold model.

The panel threshold model [33] is capable of effectively capturing structural breaks in sample data and accurately addressing nonlinear issues within economic systems. Additionally, it accounts for fixed effects during data processing, demonstrating strong performance in general panel data models. To test the threshold effect of the digital economy on the relationship between GEA and UME, as proposed in Hypothesis 2, this paper employs the dynamic threshold regression model developed by Kremer et al. (2013) [34], which builds upon Hansen’s general threshold regression framework [33]. Compared to traditional threshold regression, this method more effectively addresses endogeneity between independent and dependent variables, thereby enhancing the robustness of nonlinear estimation results. Given the uncertainty regarding the exact number of thresholds, and considering computational cost-efficiency [35,36], a single-threshold model (as shown in Equation 3) is established for testing purposes.


$$UME_{it}=\alpha_{0}+\alpha_{1}UME_{it-1}+\beta_{1}GEA_{it}\times I({DE}_{it}\leq \gamma_{1})+\beta_{2}GEA_{it}\times I(DE_{it}>\gamma_{1})+\gamma X+\mu_{i}+\varphi_{t}+\varepsilon_{it}$$ (3)

In Equation (3), I(*) denotes the indicator function, γ represents the threshold value, and DE is the threshold variable, which reflects the level of digital economy development. A key objective in the dynamic panel threshold regression model is to eliminate fixed effects or city-specific influences. To address this, we apply the fixed-effects transformation method. However, as noted by Kremer et al. [30], neither the first-difference method nor the within-transformation method can be used to remove city-specific influences, as doing so would violate the distributional assumptions [33,37]. Following the recommendation of Arellano and Bover (1995) [38], we conduct a forward orthogonal deviation transformation on the formula (3), ensuring that the error term satisfies the following transformation formula:


$$\varepsilon_{it}^{*}=\sqrt{\frac{T-t}{T-t+1}}[\varepsilon_{it}-\frac{\text{1}}{T-t}(\varepsilon_{it+1}+\cdots +\varepsilon_{iT})$$ (4)

Therefore, the orthogonal forward deviation transformation ensures that the error terms are uncorrelated, and the variance calculation adheres to the formula provided below:


$$Var(\varepsilon_{it})=\sigma^{2}I_{T};\;Var(\varepsilon_{it}^{*})=\sigma^{2}I_{T-1}$$ (5)

### 3.2. Variables

#### 3.2.1. Independent variable: Government Environmental Attention (GEA).

The annual government report serves as a critical document for governmental bodies at all levels in China, utilized to review and summarize recent accomplishments while outlining future development strategies [39]. Typically, the report encompasses various domains, such as economic growth, environmental protection, and social security. At the beginning of each year, governmental agencies submit these reports to detail the achievements of the previous year and delineate plans for the upcoming year. These reports not only consolidate societal demands and public consensus but also play a decisive role in shaping the government’s priorities for the year. The degree of emphasis placed on environmental issues within the report directly reflects the government’s commitment and the comprehensiveness of its environmental governance strategies. Local governments publish their annual reports on official websites to enhance communication between the government and the public [40].

Therefore, we employed the word frequency statistics method to calculate the frequency of terms related to ecological and environmental protection in municipal government work reports, followed by logarithmic transformation for standardization. Word frequency statistics represent a widely used approach in text analysis, involving the quantification of word occurrences within a given text. Specifically, this method computes the ratio of a word’s occurrence to the total number of words in the document, thereby reflecting the term’s relative importance and relevance. Given that government work reports may include terms associated with the general concept of “environment” (e.g., business environment, policy environment, and economic environment), which are not directly pertinent to ecological and environmental protection, we selected the following 15 keywords closely aligned with ecological issues based on prior research [41] to minimize potential interference from irrelevant terminology. The keywords used for statistical analysis include: environmental protection, ecological environment, treatment plant, ecological construction, comprehensive management, coverage rate, returning farmland to forest, pollutants, sewage treatment, green mountains and clear waters, air quality, PM2.5, garbage disposal, energy conservation, and cleanliness.

#### 3.2.2. Dependent variable: Urban Ecosystem Metabolic Efficiency (UME).

Urban metabolism can be defined as the process through which urban systems acquire material and energy inputs and generate waste outputs [1,3]. This concept is primarily derived from an analogy with the metabolic processes of living organisms [3], as cities, much like biological entities, extract resources from their surrounding environments and produce waste byproducts [2]. Urban metabolic efficiency refers to the ratio of output to input within the urban metabolic system. A higher level of metabolic efficiency signifies that the system achieves greater overall output while utilizing fewer resources, less energy, reduced human labor, and causing minimal environmental impact [42]. Research on urban metabolic systems frequently employs the Material Flow Analysis (MFA) method [43], which enables a comprehensive and systematic representation of the flows and accumulations of metabolic elements by integrating and summarizing the components of the system. This study adopts the urban material flow analysis framework, defining direct material input as the input component and categorizing the output into desired outcomes—such as economic and social development—and undesired outputs, such as environmental pollution. Within this analytical framework, the Slack-Based Measure (SBM) model, incorporating non-desired outputs (Undesirable-Output SBM), serves as an effective tool for evaluating the metabolic efficiency of urban ecosystems [8]. The SBM model can incorporate the slack variables of input and positive and negative output factors into a unified framework, thereby providing a more objective and accurate evaluation of efficiency [44]. Assuming there are n decision-making units in the production system, m units of input yield S1 units of expected output and S2 units of undesired output. Based on the production possibility set, the SBM model is established as follows:


$$\rho^{*}=\min\frac{\frac{\text{1}}{m}\sum {\mathrm{\backslash nolimits}}_{i=1}^{m}\frac{{\overset{―}{X}}_{i}}{x_{i0}}}{\frac{\text{1}}{S_{1}+S_{2}}\left(\sum {\mathrm{\backslash nolimits}}_{r=1}^{s_{1}}\frac{{\overset{―}{y}}_{r}^{g}}{y_{r_{0}}^{g}}+\sum {\mathrm{\backslash nolimits}}_{r=1}^{s_{2}}\frac{{\bar{y}}_{r}^{b}}{y_{r0}^{b}}\right)}$$ (6)

$$s.t.\left\{ \begin{array}{c} x_{0}=X\theta +S^{-},y_{0}^{g}=Y^{g}\theta -S^{g},y_{0}^{b}=Y^{b}\theta -S^{b}\\ \bar{x}\geq \sum {\mathrm{\backslash nolimits}}_{j=1,\neq 0}^{n}\theta_{j}x_{j},{\overset{―}{y}}^{g}\leq \sum {\mathrm{\backslash nolimits}}_{j=1,\neq 0}^{n}\theta_{j}y_{j}^{g},{\overset{―}{y}}^{b}\leq \sum {\mathrm{\backslash nolimits}}_{j=1,\neq 0}^{n}\theta_{j}y_{j}^{b}\\ \bar{x}\geq x_{0},{\overset{―}{y}}^{g}\leq y_{0}^{g},{\overset{―}{y}}^{b}\leq y\\ \sum {\mathrm{\backslash nolimits}}_{j=1,\neq 0}^{n}\theta_{j}=1,S^{-}\geq 0,S^{g}\geq 0,S^{b}\geq 0,{\overset{―}{y}}^{g}\geq 0,\theta \geq 0 \end{array}\right.$$ (7)

In Equations (6) and (7), $x\in R^{m},y^{g}\in R^{S_{1}},y^{b}\in R^{S_{2}}$; $\rho^{*}$ represent the efficiency values of the constraint units. Specifically, S-, Sg, and Sb denote the slack variables for input, expected output, and non-expected output, respectively. Additionally, x represents the input, yg corresponds to the expected output, and yb refers to the non-expected output.

UME measurement index system is primarily divided into three components: input, positive output, and negative output. Specifically, the input component includes: (1) water resource input, measured by total societal water consumption; (2) land resource input, indicated by urban construction land area; (3) energy input, which consists of direct energy sources such as natural gas and liquefied petroleum gas, as well as indirect energy consumption like electricity usage. To address unit inconsistencies, energy consumption is standardized into equivalent coal units, with conversion coefficients of 1.33 kg·m ⁻ ³ for natural gas, 1.7143 kg·kg ⁻ ¹ for liquefied petroleum gas, and 0.1229 kg·(kW·h)⁻¹ for electricity. (4) Labor input, quantified by the number of employees per unit. (5) Capital input, assessed using the capital stock calculated via the perpetual inventory method. Positive outputs encompass: (1) educational welfare, represented by the number of students enrolled in primary and secondary schools; (2) wage and welfare, indicated by the total wages of employed workers; (3) economic production, measured by regional GDP. Negative outputs include: (1) wastewater discharge, quantified by industrial wastewater volume; (2) exhaust gas emissions, represented by industrial sulfur dioxide emissions; (3) solid waste emissions, indicated by industrial smoke (or dust) emissions.

#### 3.2.3. Threshold variable: Digital Economy Development (DE).

The digital economy represents an emerging form of economic organization. The G20 has provided a clear definition of the digital economy in its “Initiative on Digital Economy Development and Cooperation,” which describes it as an economic system that regards digital information and knowledge as core production factors, utilizes information networks as infrastructure, and relies on ICT (Information and Communication Technology) as its primary supporting mechanism, to enhance economic efficiency and optimize industrial structures. As a relatively new economic paradigm, there is currently no universally accepted or representative indicator system for measuring the digital economy. Existing research often employs single-dimensional indicators to assess the digital economy, such as the level of development in information and communication technology and internet infrastructure [45], as well as metrics like internet penetration rates and per capita broadband access [46]. However, these singular indicators are often insufficient to capture the overall development level of the digital economy comprehensively. In response, some scholars have attempted to construct multi-dimensional indicator systems encompassing areas such as internet development and digital finance. Meanwhile, the European Union emphasizes a broader measurement framework that incorporates multiple dimensions of social development in assessing the digital economy and society [47]. Given the limited availability of city-level digital economy indicators, this study adopts the methodology proposed by Wang and Zhang (2024) [48] to construct a municipal digital economy development index based on two key dimensions: internet development level and digital transaction activity. Using the entropy weight method, this study calculates the digital economy development index for each city. Specifically, the internet development level reflects the foundational infrastructure of the digital economy, while digital transactions indicate the application and operational depth of digital technologies within urban economies [49], thereby enabling a relatively comprehensive assessment under current data constraints. The specific indicator system is outlined as follows Table 1:

**Table 1:** Indicator system of urban digital economy development.

Primary index	Secondary index	Index calculation
Internet development level	Internet penetration rate Proportion of relevant employees Related output of internet industry Mobile phone penetration level	The proportion of Internet broadband into the number of households The proportion of the number of employees in computer-related services in urban employment Per capita telecommunications service volume Number of mobile phones per capita
The digital transaction	Digital inclusive finance	Peking University Digital Inclusive Financial Index

#### 3.2.4. Control variables.

Based on the existing literature, the control variables in this study are defined as follows: Economic development level (GDP): in low-income regions, economic growth is primarily driven by expanding production activities with higher carbon dioxide emission intensities, whereas high-income regions exhibit a stronger demand for environmental quality, which influences the efficiency of urban metabolism. This variable is measured using the natural logarithm of per capita GDP. Foreign direct investment (FDI): expressed as the ratio of actual utilized foreign capital to regional gross domestic product. Urbanization (Urb): urbanization involves the concentration of population and industries, significantly impacting UME. This variable is measured as the proportion of the urban population relative to the total population. Fiscal intervention (Fac): assessed by the ratio of government general fiscal expenditure to general fiscal revenue. Industrial structure (InS): calculated as the ratio of the added value of the tertiary industry to the GDP of the city.

### 3.3. Data

The research focuses on prefecture-level and above cities in China from 2005 to 2022. Cities with incomplete data are excluded, leaving a total of 272 cities as the research subjects. Among the input-output indicators used in the calculation of UME, “water resource input” data are sourced from the China Urban Construction Statistical Yearbook, and “wage and welfare” data are obtained from the annual statistical yearbooks of individual cities; all remaining indicator data are drawn from the China Urban Statistical Yearbook. The data used in text analysis to derive GDA are collected from the Annual Government Work Reports of each city. For the calculation of DE indicators, data on “internet penetration rate” and “mobile phone penetration level” are obtained from the China Urban Statistical Yearbook; “proportion of relevant employees” and “related output of internet industry” are primarily sourced from municipal statistical yearbooks. Digital inclusive finance data is based on the Digital Inclusive Finance Index developed by Peking University. Finally, the data for variables GDP, FDI, Urb, InS, and Fac are all obtained from the China Urban Statistical Yearbook.Tables 2 and 3 respectively illustrate the data sources and processing methods for the main variables, along with the descriptive statistics of these variables.

**Table 2:** Data sources and processing methods of main variables.

Variables	Data processing method	Data sources
UME	Calculation of the SBM Model for Non-desired Outputs	China Urban Statistical Yearbook, China Regional Economic Statistical Yearbook, China Urban Construction Statistical Yearbook, and statistical yearbooks of various cities
GDA	Based on 15 environmental protection keywords, the frequency of word usage was calculated and the logarithmic value was taken.	Annual Government’s Work Report
DE	Based on the indicator system of urban digital economic development, the entropy weight method is used to calculate	China Urban Statistical Yearbook, Statistical Yearbooks of various cities, “Digital Inclusive Finance Index of Peking University”
GDP	Measured by the per capita GDP of cities and taking the logarithm.	China Urban Statistical Yearbook
FDI	the ratio of actual utilized foreign capital to regional gross domestic product	China Urban Statistical Yearbook
Urb	The proportion of urban population to the total population	China Urban Statistical Yearbook
InS	The proportion of the added value of the tertiary industry in the city’s GDP	China Urban Statistical Yearbook
Fac	the ratio of government general fiscal expenditure to general fiscal revenue	China Urban Statistical Yearbook

**Table 3:** Descriptive statistics of main variables.

Variables	Observations	Mean	Max	Min	Std. dev
UME	4896	0.3245	1	0.0062	0.2683
GDA	4896	7.5491	21.0265	0.0755	2.8046
DE	4896	0.1334	0.7132	0.0146	0.0580
GDP	4896	0. 4162	15. 7201	0. 2194	5. 3243
FDI	4896	0.0195	0. 1212	0	0.0226
Urb	4896	0.5692	0.9227	0.4163	0.2553
InS	4896	0.4626	0.8218	0.1274	0.1225
Fac	4896	2.8452	35.6716	0.7235	2.5064

## 4. Results

### 4.1. Results of spatial correlation test

#### 4.1.1. Spatial correlation analysis.

The presence of spatial autocorrelation may compromise the effectiveness of traditional statistical methods, as it violates the fundamental assumptions of independence and randomness in conventional statistics. To examine the spatial autocorrelation of UME, this study employs the spatial autocorrelation index for analysis. The spatial autocorrelation index is typically quantified using Moran’s I index, with its calculation presented in Equation (8).


$$Moran^{\prime }sI=\frac{n}{\sum {\mathrm{\backslash nolimits}}_{i}\sum {\mathrm{\backslash nolimits}}_{j}w_{ij}}\times \frac{\sum {\mathrm{\backslash nolimits}}_{i}\sum {\mathrm{\backslash nolimits}}_{j}w_{ij}\left(X_{i}-\overset{―}{X}\right)\left(X_{j}-\overset{―}{X}\right)}{\sum {\mathrm{\backslash nolimits}}_{i}{\left(X_{i}-\overset{―}{X}\right)}^{2}}$$ (8)

**Fig 2:**
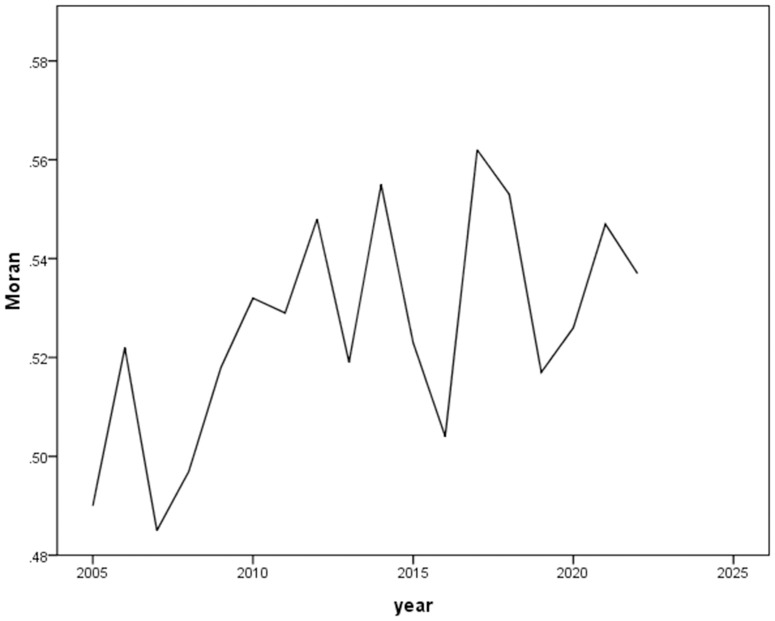
The trend of the mean value of Moran’s Iindex of UME.

In Equation (8), W is a spatial matrix, x is the variable to be tested, and $\bar{x}$ is the mean. The range of the Moran’s I index is [−1, 1]. Based on the spatial autocorrelation Moran’s I index, it was found that the mean value of Moran’s I for the metabolic efficiency of urban ecosystems during the study period was 0.526 (Fig 2), and all values passed the significance test at the 1% confidence level. It indicates that there is a significant spatial correlation in the metabolic efficiency across urban ecosystems. Therefore, considering the spatial dependence of UME is crucial in this analysis.

#### 4.1.2. Selection of spatial econometric models.

To examine the relationship between GEA and UME, significance tests including the LM test, Robust LM test, and LR test were conducted. The results of these tests were utilized to determine the most suitable spatial econometric model for analysis. According to Table 4, the LM test revealed that both the LM-Error and LM-Lag tests passed at the 1% significance level, suggesting that both the spatial error model (SEM) and spatial autoregressive model (SAR) are viable options. Additionally, the Hausman test confirmed that the fixed-effects model is the most appropriate choice. Finally, the LR and Wald tests were employed to assess whether the spatial Durbin model (SDM) should be reduced to either the SEM or SAR model. The results rejected the null hypothesis, indicating that the spatial Durbin model is the most appropriate for this analysis.

**Table 4:** Identification of spatial model test results.

	Spatial adjacency matrix		Spatial economic connection matrix	
	eigenvalue	P-value	eigenvalue	P-value
LM(lag) test Robust LM(lag) test LM(error) test Robust LM(error)test Wald_spatial_lag LR_spatial_lag Wald_spatial_error LR_spatial_error Hausman test	56.0281*** 32.4493* 1268.7354*** 1207.0036*** 118.5306*** 47.2548*** 103.3291*** 39.0063*** 124.8362***	0.0000 0.0726 0.0000 0.0000 0.0000 0.0000 0.0000 0.0000 0.0000	72.6425*** 36.7431* 1290.2846*** 1243.1958*** 127.9427*** 54.3386*** 112.4972*** 36.1439*** 126.2875***	0.0000 0.0655 0.0000 0.0000 0.0000 0.0000 0.0000 0.0000 0.0000

### 4.2. Benchmark estimation results of spatial Durbin model

Table 5 presents the benchmark estimation results of the spatial Durbin model. The estimated coefficients for GEA are 0.0076 and 0.0050, which are statistically significant at the 1% level. This indicates that GEA plays a critical role in enhancing UME, thereby confirming Hypothesis 1 (H1). The coefficients for WGEA are 0.0026 and 0.0034, which are significant at 1% and 5%, respectively. These findings suggest that GEA not only improves their own cities’ metabolic efficiency but also positively influences neighboring cities, particularly those with close economic ties. This may stem from intergovernmental interactions, where neighboring or economically interconnected cities exhibit mutual influence. Furthermore, cities with higher levels of GEA may produce more low-energy, low-emission green products, which can further impact neighboring or economically linked cities through input-output relationships [50].

**Table 5:** Benchmark estimation results of spatial Durbin model.

Variables	Spatial adjacency matrix	Spatial economic connection matrix
	(1)	(2)
GEA	0.0076*** (8.258)	0.0050*** (6.442)
GDP	0.2864*** (7.013)	0.3007*** (4.695)
FDI	−0.0488*** (−6.253)	−0.0329** (−2.146)
Urb	−0.2317*** (−5.476)	−0.2758** (−2.119)
InS	0.3925*** (4.998)	0.3379*** (6.434)
Fac	.2107 (−1.149)	−0.1468 (−1.052)
WGEA	0.0026*** (4.865)	0.0034** (2.192)
WGDP	0.0032* (1.976)	0.0026 (1.438)
WFDI	−0.3570 (−0.938)	−0.4416 (−0.857)
WUrb	−0.0429*** (−3.583)	−0.0861 (−1.292)
WInS	0.0821 (0.694)	0.0735 (1.088)
WFac	0.0558 (1.243)	0.0306 (0.721)
ρ	0.0297*** (6.548)	0.0346*** (5.902)
Fixed effect	Yes	Yes
R^2^	0.6287	0.6744

Note: *, **, and *** are significant at the significance level of 10%, 5%, and 1%, respectively, with t-statistics in parentheses.

The significantly positive coefficients of GDP and InS suggest that economic development and industrial structure have a promoting effect on UME. It implies that cities with higher levels of economic development and industrial structure tend to exhibit greater metabolic efficiency in their urban ecosystems. Conversely, the negative and statistically significant coefficients of FDI and Urb indicate that foreign direct investment does not contribute positively to improving the UME in Chinese cities, which may highlight the need for greater attention to the quality of investment attraction. Additionally, urbanization (Urb) significantly suppresses the improvement of metabolic efficiency in China’s urban ecosystems, potentially due to the relatively low quality of urbanization currently observed in China. The rapid expansion of urban populations leads to excessive resource consumption and pollution emissions, thereby hindering ecological sustainability.

### 4.3. Decomposition of spatial effects

Accurately explaining spatial spillover effects based solely on the point estimation of spatial Durbin models is challenging. A commonly employed auxiliary approach involves calculating the direct and indirect effects through partial differentiation [51,52], which enhances the interpretation of spatial spillover effects. The specific decomposition formula is as follows:


$$Y_{it}={\left(I-\rho W\right)}^{-1}\left(X_{it}\alpha +WX_{it}\theta \right)+{\left(I-\rho W\right)}^{-1}\psi_{i}+{\left(I-\rho W\right)}^{-1}\lambda_{t}+{\left(I-\rho W\right)}^{-1}\varepsilon_{it}$$ (9)

$$\frac{\partial Y}{\partial X_{it}}\times \frac{\partial Y}{\partial X_{Nt}}]=\left[\begin{array}{ccc} {}*20c\frac{\partial y_{1}}{\partial X_{1t}} & \times & \frac{\partial y_{1}}{\partial X_{Nt}}\\ \vdots & \vdots & \vdots \\ \frac{\partial y_{N}}{\partial X_{1t}} & \times & \frac{\partial y_{N}}{\partial X_{Nt}} \end{array}\right]={\left(I-\rho W\right)}^{-1}\left[\begin{array}{ccc} {}*20c\alpha_{t} & \begin{array}{cc} {}*20c\omega_{12}\lambda_{t} & \ldots \end{array} & \omega_{1N}\lambda_{t}\\ \omega_{21}\lambda_{t} & \begin{array}{cc} {}*20c\alpha_{t} & \vdots \end{array} & \omega_{2N}\lambda_{t}\\ \begin{array}{c} {}*20c\vdots \\ \omega_{N1}\lambda_{t} \end{array} & \begin{array}{cc} {}*20c\begin{array}{c} {}*20c\vdots \\ \omega_{N2}\lambda_{t} \end{array} & \begin{array}{c} {}*20c\ddots \\ \ldots \end{array} \end{array} & \begin{array}{c} {}*20c\vdots \\ \alpha_{t} \end{array} \end{array}\right]$$ (10)

By differentiating the explanatory variables in Equation (1), we derive Equation (9). Taking the partial derivative concerning the k-th explanatory variable yields the partial derivative matrix presented in Equation (10). In the matrix of Equation (10), the mean of the diagonal elements corresponds to the direct effect, whereas the mean of the off-diagonal elements reflects the indirect effect.

Table 6 presents the results of spatial effect decomposition based on the spatial adjacency matrix. The direct and indirect effects of GEA on UME are 0.0082 and 0.0027, respectively. Significance testing confirms that GEA exhibits a significant spatial spillover effect. Enhancing GEA in a local area not only improves the UME of its urban ecosystem but also positively influences the UME of adjacent cities’ ecosystems. Notably, the direct effect of GEA is considerably stronger than its indirect effect. While GEA has positive and negative impacts on neighboring cities, the overall indirect effect remains significantly positive due to the dominance of positive influences. The negative effects primarily stem from inter-city competition for talent,resources, and pollution spillovers. Yet these are outweighed by the positive contributions.

**Table 6:** Decomposition results of spatial effects.

Variables	Direct effect	Indirect effect	Total effect
	(1)	(2)	(3)
GEA	0.0082*** (6.507)	0.0027** (2.185)	0.0109*** (3.811)
GDP	0.3579*** (8.246)	0.0482*** (6.482)	0.3988*** (10.75)
FDI	−0.0524*** (−3.913)	−0.0795 (−1.226)	−0.0587*** (−4.152)
Urb	−0.2874*** (−6.822)	−0.0368* (−1.948)	−0.2963*** (−5.753)
InS	0.3432*** (4.687)	0.0591 (0.882)	0.3507*** (3.139)
Fac	0.0074 (1.356)	0.0025 (1.064)	0.0081 (1.225)

Note: *, **, and *** are significant at the significance level of 10%, 5%, and 1%, respectively, with t-statistics in parentheses.

### 4.4. Robustness test

This paper employs four approaches for robustness testing: First, the measurement method of UME is altered. Specifically, this study utilizes the Malmquist-Luenberger productivity index (ML index), which is based on a non-radial directional distance function, to derive a new dependent variable and re-estimate it. Second, the sample data are rescreened. To mitigate the influence of extreme values on the baseline regression results, the research samples are truncated at 1% and 5%, respectively, based on the dependent variable UME. Subsequently, equation (1) is regressed again, with the results reported in column (2) of Table 6. The estimation findings reveal that after excluding extreme values, the GEA coefficients continue to pass the significance test at the 1% level, aligning closely with the benchmark model’s test results. Third, high-level city samples are excluded. Given that the policy environment of separately planned cities and sub-provincial cities differs significantly from that of prefecture-level cities, this step aims to exclude potential influences of special policies within high-level city samples. Regression analysis is conducted after excluding separately planned and sub-provincial cities. Fourth, the independent variable is adjusted. In this study, the set of environmental keywords has been revised, with the total number of keywords increased to 25. The selected statistical keywords are as follows: environmental protection, green, clean, low-carbon, blue sky, clear water, green mountains, ecology, air, climate, pollution, sulfur dioxide, chemical oxygen demand, smog, particulate matter, carbon dioxide, energy consumption, raw coal, coal combustion, emissions, exhaust gas, energy conservation, emission reduction, desulfurization, and denitrification. Based on the updated keyword list, the government’s level of environmental attention has been recalculated. Table 7 presents the robustness test results under the spatial adjacency matrix, demonstrating that the signs and significance levels of the coefficients remain stable, thus confirming the robustness of the model.

**Table 7:** Robustness test.

Variables	Change the dependent variable	Test after truncation	Eliminate high-level samples	Change the independent variable
	(1)	(2)	(3)	
GEA	0.0095** (2.183)	0.0077*** (6.439)	0.0125*** (10.756)	0.0081*** (3.967)
WGEA	0.0022*** (2.590)	0.0032*** (4.046)	0.0019*** (8.005)	0.0026*** (3.714)
Control variables	Yes	Yes	Yes	Yes
Fixed effect	Yes	Yes	Yes	Yes
N	4896	4601	4554	4896
R^2^	0.6821	0.5543	0.6190	0.6382

Note: *, **, and *** are significant at the significance level of 10%, 5%, and 1%, respectively, with t-statistics in parentheses.

### 4.5. Heterogeneity test

#### 4.5.1. Test results of urban resource endowment heterogeneity.

The economic development of resource-based cities is heavily reliant on the resource industry, which exhibits significant homogeneity and low-end characteristics. Moreover, the path dependence inherent in resource-based cities during their economic growth has led to high-carbon and energy-intensive industries becoming the dominant force in urban development, resulting in substantial consumption of fossil fuels such as coal and oil. Due to excessive reliance on factor inputs, an irrational industrial structure, and low green productivity, among other issues, some resource-based cities have fallen into the “resource curse” dilemma [53,54]. In comparison to non-resource-based cities, resource-based cities may exhibit lower UME.

Cities are categorized into resource-based and non-resource-based cities for re-examination. As shown in columns (1) and (2) of Table 8, the government’s environmental concern demonstrates a more pronounced improvement effect on UME in resource-dependent cities. In contrast, for non-resource-dependent cities, the significance and magnitude of the coefficients are weaker. This disparity arises because resource-based cities heavily rely on resource industries, with high-carbon and energy-intensive industries dominating urban development. These cities exhibit low energy utilization efficiency and generate substantial undesirable outputs, such as waste gas and residue [55]. Increasing the government’s environmental concern may directly influence the core industries and their management practices in resource-based cities. The adoption of stricter environmental protection policies could compel resource extraction and processing industries to enhance energy utilization efficiency and reduce pollution emissions. Consequently, this leads to a more significant improvement in UME in resource-based cities compared to non-resource-based.

**Table 8:** Heterogeneity test (1).

Variables	Resource-based city	Non-resource-based city	High GDP per capita city	Low GDP per capita group
	(1)	(2)	(3)	(4)
GEA	0.0163*** (5.214)	0.0025*** (3.436)	0.0034*** (3.829)	0.0102*** (6.770)
WGEA	0.0031** (2.007)	0.012 (1.487)	0.0017 (1.342)	0.0020* (1.906)
Control variables	Yes	Yes	Yes	Yes
Fixed effect	Yes	Yes	Yes	Yes
N	1728	3168	1638	3258
R^2^	0.612	0.584	0.636	0.629

Note: *, **, and *** are significant at the significance level of 10%, 5%, and 1%, respectively, with t-statistics in parentheses.

#### 4.5.2. Heterogeneity test of urban economic development level.

The more developed an economy is, the stronger its demand for environmental protection tends to be [56]. Consequently, differences in economic development levels across cities may influence the relationship between GEA and the UME. To examine this heterogeneity, we classify cities into two groups based on their per capita GDP: the top one-third constitutes the high per capita GDP group, while the remaining two-thirds form the low per capita GDP group. As shown in columns (3) and (4) of Table 8, the government’s environmental concern has a more pronounced positive effect on UME in the low per capita GDP group compared to the high per capita GDP group. This disparity could stem from the fact that less economically developed cities often exhibit lower-end economic structures, lower energy utilization efficiency, and higher pollution emissions. In such contexts, enhancing GEA may lead to more significant reductions in pollution and improvements in resource utilization efficiency, thereby contributing to greater increases in UEM.

#### 4.5.3. Results of heterogeneity test on urban geographical location.

There are substantial regional disparities in China. The eastern coastal areas are relatively more developed, while the central and western regions remain relatively underdeveloped. In terms of environmental pollution, significant variations also exist among the eastern, central, and western regions [57]. To examine the impact of the regional differences in how GEA on UME, cities were categorized into eastern, central, and western groups based on their geographical locations, and the analysis was re-conducted. As shown in Table 9, in central cities, the GEA coefficient is 0.0129 and statistically significant at the 1% level. However, the GEA coefficients for western and eastern cities are 0.0082 and 0.0054, respectively, with no statistical significance observed in eastern cities. This indicates that GEA has a pronounced positive effect on UME in central and western cities, particularly in central cities. The primary reason is that the industrial structure in central cities and western cities tends to be less advanced, leading to more severe pollution challenges. However, these regions also possess greater potential for improving UME. Enhanced GEA can more effectively drive industrial upgrading, provide stronger support for green technological innovation, and promote optimal resource allocation [58], thereby significantly enhancing UME.

**Table 9:** Heterogeneity test (2).

	Eastern city (1)	Central city (2)	Western city (3)
GEA	0.0054 (1.363)	0.0129*** (6.427)	0.0082** (2.177)
W*GEA	0.0032 (0.595)	0.0017 (1.483)	0.0040 (1.139)
Control variables	Yes	Yes	Yes
Fixed effect	Yes	Yes	Yes
N	1800	1782	1314
R^2^	0.687	0.701	0.655

Note: *, **, and *** are significant at the significance level of 10%, 5%, and 1%, respectively, with t-statistics in parentheses.

### 4.6. Threshold effect test of digital economy development

Table 10 presents the test results with threshold variables set as digital economy development (DE)and its two sub-dimensions: internet development (DE1) and digital transactions (DE2). The results indicate that all three indicators successfully passed the single-threshold test, with F-statistics of 19.7705, 15.9582, and 17.3627, respectively. However, none of the indicators passed the double-threshold or triple-threshold tests. Consequently, it can be inferred that there is a significant single-threshold effect of digital economy development and its two sub-dimensions (internet development and digital transactions) on the relationship between GEA and UME.

**Table 10:** Threshold Effect Test(1).

threshold Variable	Number of thresholds	F statistic value	P value	Estimated value	Interval of confidence (α = 95%)
DE	Single threshold Double threshold Three thresholds	19.7705 12.9126 9.8472	0.0002 0.1145 0.1894	0.3154	(0.3079, 0.3226)
DE1	Single threshold Double threshold Three thresholds	15.9582 11.2612 8.0064	0.0076 0.1328 0.2490	0.2672	(0.2509, 0.2818)
DE2	Single threshold Double threshold Three thresholds	17.3627 12.4857 6.5906	0.0136 0.1074 0.1836	0.4063	(0.3975, 0.4156)

Table 11 presents the dynamic threshold effect of digital economy development on the relationship between GEA and UME. The threshold variables in columns (1), (2), and (3) are Digital Economy Development (DE), Internet Development Level (DE1), and Digital Transactions (DE2), respectively. Specifically, column (1) reveals that when DE is below the threshold value of 0.3154, the coefficient β1 equals 0.0034 and is significant at the 10% level. Conversely, when DE exceeds this threshold, the coefficient β2 increases to 0.0087 and becomes significant at the 1% level. These findings suggest that in cities with relatively low levels of digital economy development, the positive impact of GEA on UME is weaker and less pronounced. However, once the digital economy development surpasses the threshold, the promoting effect of GEA on UME becomes significantly stronger, thereby validating Hypothesis 2 (H2).

**Table 11:** The results of threshold effect of digital economy development(1).

	(1)	(2)	(3)
LagGEA	0.0054*** (5.3630)	0.0129*** (6.4285)	0.0082** (2.1772)
β1(DE≤0.3154)	0.0034* (1.9836)		
β2(DE>0.3154)	0.0071*** (8.5983)		
β1(DE1≤0.2762)		0.0058*** (4.2614)	
β2(DE1>0.2762)		0.0091*** (6.2553)	
β1(DE2≤0.4063)			0.0042 (1.2370)
β2(DE2>0.4063)			0.0067*** (3.4925)
Control variables	Yes	Yes	Yes
N	4896	4896	4896
AR-1 P-Value	−3.3572 [0.000]	−4.0886 [0.000]	−3.6243 [0.000]
AR-2 P-Value	−0.6319 [0.508]	−0.5480 [0.432]	−0.5523 [0.469]
Sargan Test P-Value	45.643 [0.391]	47.882 [0.254]	43.059 [0.481]

Note: *, **, and *** are significant at the significance level of 10%, 5%, and 1%, respectively, with t-statistics in parentheses.

The results in column (2) indicate that when DE1 is below the threshold value of 0.2762, the coefficient is significant at the 1% level, with β1 equaling 0.0058. In contrast, when DE1 exceeds the threshold value, the coefficient β2 increases to 0.0091 and remains significant. It suggests that the positive impact of GEA on UME is relatively limited when the development level of the Internet is below the threshold. However, once the Internet development level surpasses the threshold, the positive effect of GEA on UME becomes substantially stronger. The results in column (3) reveal that when DE2 is below the threshold value of 0.4063, the coefficient β1 equals 0.0042 but lacks statistical significance. Conversely, when DE2 exceeds the threshold value, the coefficient β2 rises to 0.0067 and achieves significance at the 1%. These findings demonstrate that GEA does not significantly influence UME when digital transactions are below the threshold. Nevertheless, when digital transactions exceed the threshold, GEA exhibits a pronounced and statistically significant positive effect on UME.

Although the previous analysis has uncovered the dynamic threshold effect of the digital economy (DE) on the relationship between green energy adoption (GEA) and urban metabolic efficiency (UME), this observed effect may be closely tied to the industrial structure of cities. For instance, cities predominantly reliant on manufacturing typically exhibit both lower levels of digital economic development and reduced urban metabolic efficiency. In contrast, cities hosting high-end industries often demonstrate simultaneously higher levels of digital economy and improved urban metabolic efficiency. This pattern suggests that both the digital economy and urban metabolic efficiency could be jointly influenced by common structural factors—most notably, the city’s industrial foundation. Consequently, such correlations may not accurately reflect the independent impact of the digital economy. To address this issue, we further classify the sample cities into two groups: high-end industry cities and traditional manufacturing cities. High-end industries refer to strategic emerging sectors driven by technological innovation, which generate high added value through technological advancement and industrial upgrading. Therefore, a city’s capacity for technological innovation can serve as an indicator of its level of high-end industrial development. Based on the China Urban Technological Innovation Development Report 2020 published by the Chinese Academy of Social Sciences, we identify the top 50 cities in terms of technological innovation capability as high-end industry cities, with the remaining categorized as traditional manufacturing cities. We then re-estimate the model using the dynamic panel threshold regression approach.

Table 12 presents the threshold effect test results after classifying cities into two groups. The findings indicate that both groups passed the single threshold test but failed to meet the criteria for the double and triple threshold tests. Notably, the threshold value for digital economic development in high-end industrial cities is relatively low, whereas it is significantly higher in traditional manufacturing cities. As shown in Table 13, which reports the dynamic threshold effect of DE, when the level of DE exceeds the threshold, GEA exerts a more pronounced positive impact on UME in both groups. This effect is particularly evident in traditional manufacturing cities. Specifically, when the DE is below the threshold of 0.3376, the coefficient β1 is 0.0029 and statistically significant at the 10% level. However, once DE surpasses the threshold, the coefficient β2 increases to 0.0085 and becomes significant at the 1% level. These findings suggest that traditional manufacturing cities typically have a weaker industrial foundation and a less developed digital economic environment, resulting in lower levels of UME. Consequently, the threshold for the influence of DE on the relationship between GEA and UME is relatively high. Nevertheless, once this threshold is crossed, the effect of GEA on UME becomes significantly stronger.

**Table 12:** Threshold Effect Test (2).

Cities Grouping	threshold Variable	Number of thresholds	F statistic value	P value	Estimated value	Interval of confidence (α = 95%)
High-end industrial cities	DE	Single threshold Double threshold Three thresholds	18.4580 13.6459 8.9673	0.0000 0.1739 0.2884	0.2754	(0.2679, 0.3026)
Traditional manufacturing cities	DE	Single threshold Double threshold Three thresholds	21.2681 14.5702 10.9438	0.0000 0.1678 0.1054	0.3376	(0.3298, 0.3415)

**Table 13:** The results of threshold effect of digital economy development (2).

	High-end industrial cities(1)	Traditional manufacturing cities(2)
LagGEA	0.0087 (1.193)	0.0129*** (6.4285)
β1(DE≤0.2754)	0.0036*** (7.282)	
β2(DE>0.2754)	0.0051*** (6.781)	
β1(DE≤0.3376)		0.0029* (1.974)
β2(DE>0.3376)		0.0085*** (8.566)
Control variables	Yes	Yes
N	900	3996
AR-1 P-Value	−3.6273 [0.000]	−3.2148 [0.000]
AR-2 P-Value	−0.6787 [0.721]	−0.5933 [0.640]
Sargan Test P-Value	46.579 [0.326]	44.281 [0.417]

Note: *, **, and *** are significant at the significance level of 10%, 5%, and 1%, respectively, with t-statistics in parentheses.

## 5. Discussion, conclusions and policy implications

### 5.1. Discussion

A city functions as an ecosystem with metabolic characteristics [59–61]. UME plays a critical role in determining a city’s capacity for sustainable development [62–64]. Therefore, investigating the factors influencing UME is essential for fostering their development. GEA reflects the extent to which urban governments prioritize environmental protection, potentially affecting the input-output dynamics of urban ecosystems and consequently influencing metabolic efficiency. However, existing literature has paid limited attention to the impact of informal environmental regulations, such as GEA, on the UME.

This research aims to explore the impact of GEA on UME, analyze its underlying mechanisms, examine its heterogeneity across different contexts, and propose a novel approach to enhance the role of GEA in improving UME. This study addresses a gap in the existing literature, which has paid limited attention to how GEA influences UME. The findings contribute new insights into understanding how GEA can facilitate high-quality development in developing countries. Furthermore, this research holds positive implications for expanding the scope of studies on UME and deepening the comprehension of digital economy development.

The conclusion of this article, which indicates that GEA positively affects UME, aligns with the findings [65–68]. Specifically, environmental concern encourages governments and the public to allocate more resources toward addressing environmental issues, fostering green behaviors, and ultimately promoting green development at the enterprise or regional level. This study’s approach to measuring UME enriches the literature on urban metabolism as a tool for evaluating cities’ contributions to sustainable development [69,70]. Furthermore, this research highlights the spatial spillover effects of UME, a characteristic rooted in the interconnectedness of cities—a dimension often overlooked in urban metabolism studies. This finding supports the perspective presented by Verma et al.(2025) [71].

The findings of this paper concerning the threshold effect of digital economic development on GEA and UME strongly corroborate the conclusion drawn by Teng et al.(2025) [72] that Internet utilization affects environmental behavior, thereby influencing environmental concern. Additionally, these findings align with the discovery by Li and Yang(2025) [73] that advanced digital infrastructure enhances public environmental awareness, leading to a reduction in atmospheric pollution emissions. This provides an important insight: to effectively leverage the role of informal environmental regulations in enhancing UME, certain enabling conditions must be in place. For example, the digital economy must reach a sufficient level of development, which allows informal environmental regulations to contribute to strengthening environmental governance, raising public environmental awareness, and promoting urban green innovation capabilities. It offers an effective strategy for governments to strengthen the social impact of green development concepts.

This study also has several limitations, which are primarily reflected as follows: (1) Although variations in the implementation of environmental protection policies by enterprises, the administrative capacity of local governments, and the behavioral responses of market participants may all influence the effect of GEA on UME, data availability constraints prevent a detailed examination of these factors within the heterogeneity analysis. (2) There are certain limitations in the measurement of key variables, which may partially affect the robustness of the empirical results. For example, in measuring GEA, differences in both the quantity and content of keywords related to ecological and environmental protection can lead to considerable fluctuations in the resulting GEA scores. Regarding the measurement of UME, variations in the selection of input indicators, desired outputs, and undesired outputs may also influence the calculated UME values. Moreover, current methodologies do not fully account for interdepartmental coordination within cities or the potential impact of government policies. These measurement-related limitations may somewhat constrain the interpretation of the findings.

### 5.2. Conclusion

UME is a critical indicator for assessing the sustainable development of cities as ecological systems. This paper utilizes data from Chinese cities and, based on the measurement of GEA using the text method, first investigates the spatial impact of GEA on UME through the spatial Durbin model. Subsequently, it examines the threshold effect of digital economic development on the relationship between GEA and UME by employing the dynamic panel threshold model, which can simultaneously evaluate both the threshold and endogenous effects among variables. The findings are summarized as follows: First, the results of the spatial Durbin model reveal that each city’s GEA not only contributes to its own UME improvement but also enhances the UME of neighboring cities and those with strong economic ties via spatial spillover effects. Various robustness tests confirm the presence of this positive effect. Second, heterogeneity analysis shows that in resource-based cities, cities with lower economic development levels, and central cities, the local impact and spatial spillover effects of GEA on UME are more pronounced. Third, there exists a nonlinear relationship between GEA and UME, with digital economic development acting as a threshold. When the level of digital economic development is low, the promoting effect of GEA on UME is limited; however, once digital economic development surpasses the threshold value, the promoting effect of GEA on UME becomes significantly stronger. Traditional manufacturing cities have a higher threshold for digital economic development compared to high-end industry cities. However, once the digital economy threshold is crossed, GEA plays a significantly more prominent role in UME.

### 5.3. Policy Implications

The policy implications derived from this study are as follows: Firstly, from the government perspective, municipal governments should increase their focus on environmental issues and proactively develop robust and sustainable development strategies. These strategies should include establishing stricter environmental protection regulations, offering tax incentives, and fostering inter-departmental collaboration to ensure precise resource allocation for environmental protection industries and projects. Additionally, these measures aim to encourage enterprises to engage in green technological innovation, thereby promoting efficient resource utilization and reducing waste. Continuous efforts in energy conservation and emission reduction are also essential to enhance UME. The central government must consistently prioritize environmental protection and green development at the national level to motivate local governments to maintain a strong focus on environmental sustainability. From the enterprise perspective, businesses should actively align with government green development policies and integrate them into their strategic planning and operational practices.

Secondly, fostering inter-city environmental protection cooperation and resource sharing is a critical approach to enhancing UME. The government can facilitate this process by promoting collaboration among cities in environmental protection and resource management. Cross-regional environmental protection cooperation not only contributes to reducing pollution emissions but also improves the effectiveness of environmental governance, thereby enhancing UME at a broader scale. Consequently, the government should actively encourage cities to establish cooperative partnerships and work together to address challenges related to environmental pollution and sustainable development.

Thirdly, when the government leverages GEA to enhance UME, it should develop targeted and differentiated policies tailored to the specific conditions of each city. In particular, for cities in central and western regions, resource-based cities, and those with relatively lower levels of economic development, the government should place greater emphasis on environmental protection by strengthening environmental regulations, promoting industrial restructuring and upgrading, increasing investment in green technology research and development, and optimizing the allocation of resources. Additionally, the government could consider offering subsidies and tax incentives to enterprises engaged in green technological innovation, thereby enhancing UME more effectively in these regions.

Finally, it is essential to advance the construction of digital infrastructure, including gigabit optical networks, industrial internet, big data centers, and intelligent computing centers, to expand the scale of digital economy development. Local governments, particularly those in cities where traditional manufacturing serves as the core economic pillar, should integrate digital information resources across departments to establish digital platforms for environmental information disclosure and management, ensuring real-time data updates and transparency. It will enhance public awareness of environmental issues, stimulate public participation in environmental governance, and improve the quality of environmental regulations. Furthermore, promoting the integration of digital technologies with local enterprise development and leveraging the digital economy can facilitate the transformation and upgrading of local industries. By optimizing the layout of the digital industry and providing technical support for regional green innovation, a synergy between digitalization and green development can be achieved, thereby promoting efficient resource utilization and driving the improvement of UME with greater momentum.
